# Comparison of MOLES and Mel*AI*noma for Differentiating Small Choroidal Melanomas from Nevi

**DOI:** 10.3390/cancers18050818

**Published:** 2026-03-03

**Authors:** Katerina Stripling, Hannah Coudé Adam, Mats Holmström, Gustav Stålhammar

**Affiliations:** 1 Department of Clinical Neuroscience, Division of Eye and Vision, Karolinska Institutet, 171 77 Stockholm, Sweden; 2 St. Erik Eye Hospital, 171 64 Stockholm, Sweden; 3 Eyedentity AB, 139 37 Stockholm, Sweden; mats.holmstrom@eyedentity.ai

**Keywords:** choroidal melanoma, uveal melanoma, choroidal nevus, Mel*AI*noma, MOLES, artificial intelligence, deep learning, fundus photography, ocular oncology

## Abstract

**Background:** Early identification of small choroidal melanomas is important, as metastatic risk increases with tumor size. However, distinguishing small melanomas from benign choroidal nevi is challenging and may lead to unnecessary referrals and overtreatment. Both the MOLES scoring system and the deep learning algorithm Mel*AI*noma have been developed to support assessment of pigmented choroidal lesions in non-expert settings. This study aims to compare the association between MOLES and Mel*AI*noma scores and to assess their relative association with expert melanoma versus nevus diagnosis. **Methods:** In this retrospective cohort study, 86 patients with small pigmented choroidal lesions (29 melanomas and 57 nevi) diagnosed at a national ocular oncology referral center were included. MOLES scores were assigned by ocular oncologists based on multimodal examination, whereas Mel*AI*noma scores were generated solely from color fundus photographs. Associations between scores were assessed using linear regression and the Jonckheere–Terpstra test. Univariable and multivariable binary logistic regression was used to evaluate associations with melanoma diagnosis. **Results:** Mel*AI*noma scores increased monotonically with higher MOLES categories (*p* = 0.0001). Linear regression showed a statistically significant association between MOLES and Mel*AI*noma scores, but with substantial dispersion (R^2^ = 0.16). In univariable logistic regression, both MOLES and Mel*AI*noma scores were associated with increased odds of melanoma diagnosis. Mel*AI*noma showed a stronger association with diagnosis than MOLES (R^2^ = 0.38 vs. 0.27). In multivariable analysis including both scores, each remained independently associated with melanoma diagnosis. **Conclusions:** Both MOLES and Mel*AI*noma are effective for differentiating small choroidal melanomas from nevi. Although the scores are statistically associated, they capture partly distinct information. Mel*AI*noma demonstrates slightly stronger association with melanoma diagnosis and provides fully reproducible output, supporting its role as a complementary aid in lesion triage.

## Simple Summary

Small eye melanomas can be difficult to distinguish from harmless pigmented spots in the back of the eye, called choroidal nevi. Because the risk of cancer spread increases as melanomas grow, early and accurate identification is important, but unnecessary referrals and treatment should also be avoided. Tools that support non-expert clinicians in deciding which lesions need specialist evaluation are therefore increasingly important. In this study, we compared two such tools: the MOLES scoring system, which is manually applied using several eye imaging methods, and Mel*AI*noma, an artificial intelligence system that analyzes a single color photograph of the eye. We examined how closely the two methods agree and how well each is associated with expert diagnosis of melanoma. Both tools were useful, but they captured partly different information. Mel*AI*noma showed a slightly stronger association with diagnosis despite using less imaging data, suggesting a potential role as a complementary aid for lesion triage.

## 1. Introduction

The risk of metastasis in uveal melanoma is strongly correlated with tumor size [1,2,3]. Accurate identification of melanomas when they are as small as possible could therefore be one of the most effective ways to improve survival in this aggressive disease [4,5,6], with 10-year metastasis rates ranging from 6 to 51% for tumors 0–1 mm versus >10 mm in thickness, respectively [1]. However, small choroidal melanomas may be difficult to distinguish from benign pigmented choroidal lesions, which are common in the general population and affect more than 5% of individuals of Caucasian descent [7,8,9]. Efforts to detect and treat choroidal melanomas at an early stage may therefore result in overdiagnosis and treatment of harmless lesions with potentially severe consequences for visual function [10,11,12]. At the same time, delayed referral or misclassification of early melanoma as a benign lesion can postpone treatment in patients who do require prompt specialist care [13,14].

In Sweden, opticians frequently use optical coherence tomography (OCT) and fundus photography in routine practice [15]. OCT is a noninvasive cross-sectional imaging technique of the retina and choroid that can help detect lesion-associated findings such as subretinal fluid [16,17]. Together with widespread screening for diabetic retinopathy and frequent examinations or treatment for age-related macular degeneration (AMD) or cataract, this has led to detection of an increasing number of incidental pigmented choroidal lesions [18]. As a consequence, a growing number of patients are referred to ocular oncologists, including many false-positive cases. This may pose a challenge, as ocular oncology is a highly specialized field with limited resources, and excessive referrals may delay assessment of patients who truly require intervention.

To support triage of pigmented choroidal lesions, we have developed a deep learning algorithm, Mel*AI*noma, which estimates the probability that a lesion represents a melanoma based on fundus photographs [19]. The intended use is decision support for clinicians who assess pigmented choroidal lesions with varying frequency and expertise, including opticians, optometrists, general ophthalmologists, and subspecialized ocular oncologists. By providing a reproducible photo-based risk estimate, such support may help reassure patients with benign lesions, facilitate accurate detection and referral of the smaller subset of melanomas, and reduce unnecessary referrals and associated resource use in the healthcare system. In prior evaluations, including testing on independent cohorts with an external test set, Mel*AI*noma achieved higher diagnostic accuracy than ocular oncologists, consultant ophthalmologists, and resident ophthalmologists when classifying fundus images, using multimodal expert assessment as reference [19]. When applied by opticians and optometrists, Mel*AI*noma quadrupled the odds of correctly referring a melanoma, reduced false-positive referrals tenfold, and provided net clinical benefit compared with unaided triage [15].

In contrast, the MOLES system is a well-established, manually applied scoring system designed to support non-expert monitoring of pigmented choroidal lesions and referral decisions [20,21]. MOLES is widely used by opticians and ophthalmologists, and was developed to provide a structured assessment based on five features associated with choroidal melanoma (Mushroom shape, Orange pigment, Large size, Enlarging tumor, and Subretinal fluid), each scored as absent, borderline, or present, and to translate this into risk categories with corresponding management recommendations [20,21]. By assessing and grading five visual risk factors for growth, lesions are classified as common nevus, low-risk nevus, high-risk nevus, or probable melanoma [21]. Several studies have evaluated MOLES in ocular nevus clinics and tertiary referral cohorts, generally reporting high sensitivity for identifying lesions requiring specialist assessment [20,21,22,23,24]. Unlike Mel*AI*noma, MOLES relies on examiner recognition of clinical and imaging features and may therefore be influenced by observer experience and available imaging modalities. Here, MOLES was scored by subspecialized ocular oncologists to benchmark the manual system against experienced graders, reducing the risk that any difference versus Mel*AI*noma could be attributed to limited familiarity with MOLES among non-expert users; we have evaluated MOLES performance in optician/optometrist triage separately [15].

In the present study, we compare MOLES scores assigned by ocular oncologists with Mel*AI*noma scores generated from fundus photographs. Our aims are to examine whether MOLES and Mel*AI*noma scores correlate and to assess which of the two is more strongly associated with expert classification of choroidal melanoma versus choroidal nevus.

## 2. Materials and Methods

### 2.1. Patients and Lesions

This study included a cohort of 86 patients with one pigmented choroidal lesion each, diagnosed as either small choroidal melanoma or choroidal nevus at St. Erik Eye Hospital, Stockholm, Sweden. This study was approved by the Swedish Ethical Review Authority (reference 2025-05054-01) and adhered to the tenets of the declaration of Helsinki. None of the included lesions underwent biopsy or histopathologic confirmation; diagnoses were based on clinical multimodal assessment by subspecialized ocular oncologists.

Inclusion criteria were as follows:

1. Age >18 years at the time of examination.
2. Fundus photograph obtained after 1 January 2010, corresponding to a period when medical records were fully digitalized, facilitating reliable assessment of follow-up.
3. Diagnosis of either choroidal melanoma (International Classification of Diseases, 10th Revision [ICD-10] code C69.3) or choroidal nevus (ICD-10 code D31.3).
4. Diagnosis established by a subspecialized ocular oncologist.
5. For lesions classified as nevi at the time of photography, a minimum of 5 years of follow-up without reclassification as melanoma was required. Lesions that were diagnosed as melanoma at a later time point (e.g., due to documented growth) were classified as melanomas in the present study. This criterion was applied to facilitate detection of early signs of malignancy at a stage when small melanomas are difficult to distinguish from nevi.

Exclusion criteria were:

1. Fundus photographs of insufficient quality, where factors such as poor focus, motion artifacts, over- or underexposure, or reflections prevented reliable assessment of lesion extent or the presence of features such as orange pigment or drusen. Minor image imperfections that did not impede assessment (e.g., focal blur or limited overexposure) were not sufficient for exclusion.
2. Photographs in which less than half of the lesion was visible, acknowledging the limitation in accurately estimating the extent of the non-visible portion.
3. Lesions obscured by retinal detachment, vitreous hemorrhage, or similar conditions.

The included lesions were imaged using either a standard-field fundus camera (45° field of view; Canon Medical Systems Europe B.V., Amstelveen, The Netherlands; *n* = 55) or an ultra-widefield camera (pseudocolor images covering 200°; Optos Inc., Dunfermline, Scotland; *n* = 31). This cohort has been described in detail previously, including the multimodal diagnostic workup used by ocular oncologists, as it was employed as a test cohort during the development and validation of the Mel*AI*noma algorithm [19]. The cohort was not used for development or training of the Mel*AI*noma deep learning model.

### 2.2. MOLES

MOLES is a clinical scoring system based on five features: Mushroom shape, Orange pigment, Large size, Enlarging tumor, and Subretinal fluid. Each feature is assigned a score of 0, 1, or 2, reflecting absence, borderline presence, or definite presence [20,21]. Mushroom shape is highly suggestive of choroidal melanoma and is included to ensure referral even in absence of other suspicious features. Orange pigment has been identified as an important risk factor for growth of melanocytic lesions, despite occurring in other choroidal conditions [25,26]. Large size is included because most choroidal nevi are small; in population-based studies, the mean largest basal diameter is approximately 1.25 mm, only a minority exceed 5.5 mm, and increased thickness is associated with a higher risk of future growth [27]. Although choroidal nevi may enlarge slowly over an extended period, more rapid growth is suggestive of malignant transformation [28]. Subretinal fluid is also included, as it has consistently been associated with lesion growth. Based on the total score, lesions are categorized as common nevus (score 0), low-risk nevus (score 1), high-risk nevus (score 2), or probable melanoma (score > 2) [21]. In this study, all MOLES scores were assigned by ocular oncologists at St. Erik Eye Hospital, with access to multimodal imaging for each lesion to assess the presence of risk factors, such as OCT for detection of subretinal fluid and autofluorescence for identification of orange pigment. In contrast, Mel*AI*noma scores were based solely on analysis of color fundus photographs of the lesion.

### 2.3. Statistical Analyses

Associations between MOLES score and Mel*AI*noma score were evaluated using linear regression. As MOLES score is an ordinal variable and Mel*AI*noma score is continuous, an ordered trend was additionally assessed using the Jonckheere–Terpstra test, which does not assume normality. To evaluate the association between each score and diagnostic outcome, binary logistic regression was performed with true diagnosis (melanoma vs. nevus) as the dependent variable. Odds ratios (ORs) with 95% confidence intervals (CI) were reported. Mel*AI*noma score and MOLES score were also entered simultaneously as predictors in a multivariable logistic regression model. No interaction terms or variable transformations were included. Model fit was assessed using the Akaike Information Criterion corrected for small sample size (AIC) and by evaluation of calibration with the Hosmer–Lemeshow test. Mel*AI*noma scores were generated by loading fundus photographs into the Mel*AI*noma software (v0.1; Eyedentity AB, Stockholm, Sweden), which outputs a probability between 0 and 1 that the lesion represents a melanoma. All statistical tests were two-sided, except the Jonckheere–Terpstra test, which was prespecified as one-sided to test for an increasing trend of Mel*AI*noma scores with higher MOLES categories; a significance level of *p* < 0.05 was used. Data management and statistical analyses were performed using R v4.4.3 (R Foundation for Statistical Computing, Vienna, Austria) and GraphPad Prism v10.6.1 (GraphPad Software, San Diego, CA, USA).

## 3. Results

### 3.1. Descriptive Statistics

The study included 86 patients with 86 pigmented choroidal lesions, of whom 48 (56%) were female. Of the 86 lesions, 57 (66%) were diagnosed as nevi and 29 (34%) as melanomas. Compared with nevi, melanomas had a greater mean thickness (2.8 vs. 1.6 mm, Welch’s two-sample *t*-test *p* < 0.001) and larger mean LBD (6.6 vs. 4.4 mm, *p* < 0.001). Further cohort characteristics are summarized in Table 1.

### 3.2. Distribution of MelAInoma Scores Across MOLES Scores

**Figure 1:**
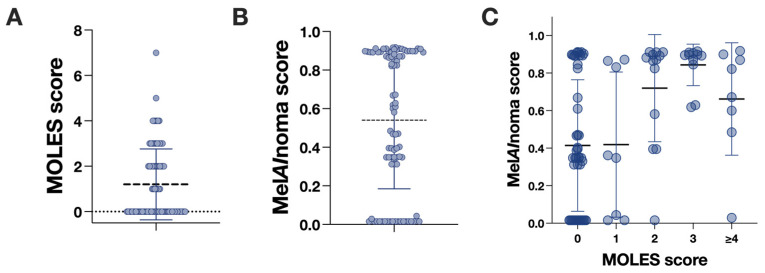
Distribution of MOLES and Mel*AI*noma scores. Each point represents one lesion. (**A**) Distribution of MOLES scores across the included lesions, shown with horizontal jitter to reduce overlap. The horizontal line indicates the mean, and whiskers indicate the standard deviation (SD). (**B**) Distribution of Mel*AI*noma scores across the included lesions. (**C**) Distribution of Mel*AI*noma scores stratified by MOLES score (0, 1, 2, 3, and ≥4). Horizontal lines indicate the mean, and error bars indicate one SD.

Mel*AI*noma scores increased monotonically with increasing MOLES categories. The distribution of lesions by MOLES score was as follows: MOLES 0 (*n* = 46), MOLES 1 (*n* = 8), MOLES 2 (*n* = 13), MOLES 3 (*n* = 11), and MOLES ≥ 4 (*n* = 8). Using the Jonckheere–Terpstra test to evaluate an ordered trend, there was strong evidence for increasing Mel*AI*noma scores with higher MOLES categories (JT = 1620, *p =* 0.0001, one-sided test for increasing trend). This association persisted despite substantial ties in Mel*AI*noma score values and unequal group sizes, indicating a consistent monotonic relationship between MOLES score and AI-based risk estimation (Figure 1). In addition, a rank-based one-way ANOVA (Kruskal–Wallis test) showed differences in Mel*AI*noma score distributions across MOLES categories (*p* = 0.0008).

### 3.3. Linear Regression

**Figure 2:**
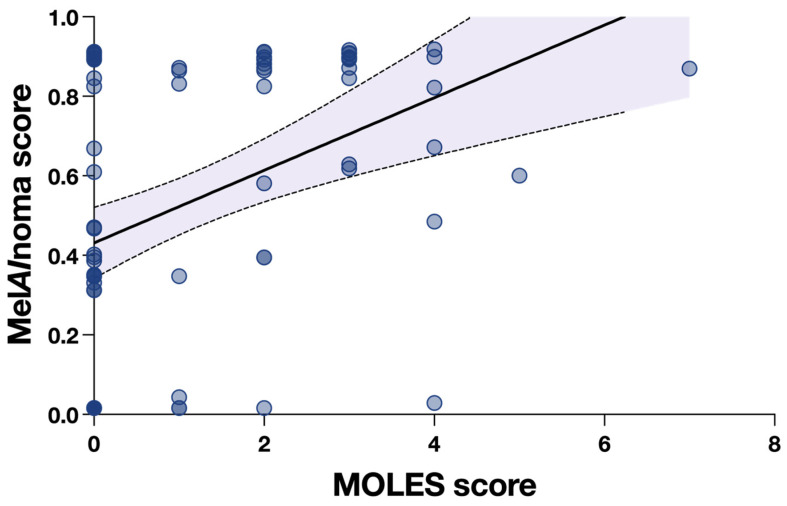
Association between MOLES score and Mel*AI*noma score. Scatter plot showing Mel*AI*noma score as a function of MOLES score. The solid line represents the fitted linear regression (slope 0.09 per MOLES unit; *p <* 0.0001), and the shaded area indicates the 95% confidence interval (CI).

In linear regression analysis, increasing MOLES score was associated with higher Mel*AI*noma score (slope 0.09 per MOLES unit, 95% CI 0.05 to 0.14, *p* < 0.0001). However, the association showed considerable dispersion, with MOLES score explaining only 16% of the variability in Mel*AI*noma score (R^2^ = 0.16, Figure 2).

### 3.4. Binary Logistic Regression for MOLES

**Figure 3:**
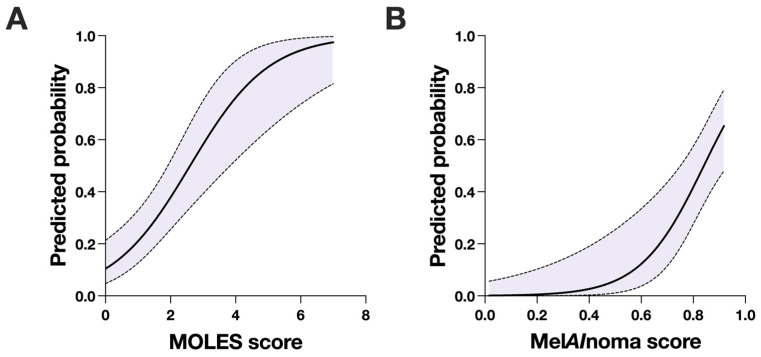
Binary logistic regression models. (**A**) Predicted probability of melanoma as a function of MOLES score from a binary logistic regression model. (**B**) Predicted probability of melanoma as a function of Mel*AI*noma score from a binary logistic regression model. In both panels, the solid line represents the fitted model, and the shaded area indicates the 95% confidence interval (CI).

In binary logistic regression with MOLES score as the sole predictor, higher MOLES score was associated with increased odds of melanoma diagnosis (Figure 3A). Each one-unit increase in MOLES score was associated with a more than twofold increase in the odds of melanoma (OR 2.29, 95% CI 1.59 to 3.50, *p* < 0.0001, Table 2). Model fit was acceptable, with a Tjur’s R^2^ of 0.27.

### 3.5. Binary Logistic Regression for MelAInoma

In binary logistic regression using Mel*AI*noma score as the sole predictor, higher Mel*AI*noma score was associated with increased odds of melanoma diagnosis (Figure 3B). When scaled per 0.1-unit increase, the odds of melanoma increased by a factor of 2.27 (OR 2.27 per 0.1 increase, 95% CI 1.57 to 4.23, *p* < 0.0001, Table 3). Compared to the MOLES model, this model explained a larger proportion of outcome variability (Tjur’s R^2^ = 0.38).

### 3.6. Multivariable Binary Logistic Regression

In multivariable binary logistic regression including both MOLES score and Mel*AI*noma score, both predictors remained associated with melanoma diagnosis. After adjustment for MOLES score, each 0.1-unit increase in Mel*AI*noma score was associated with a 2.82-fold increase in the odds of melanoma (OR 2.82, 95% CI 1.62 to 6.85, *p* < 0.0001, Table 4). MOLES score also remained associated with melanoma diagnosis after adjustment for Mel*AI*noma score (OR 2.24 per one-unit increase, 95% CI 1.40 to 4.02, *p* = 0.002). The combined model showed substantially improved the fit compared with the intercept-only model (AIC 55.3 vs. 103.2), and there was no evidence of poor calibration based on the Hosmer–Lemeshow test (*p* = 0.86).

### 3.7. Bootstrap Internal Validation of the Multivariable Prediction Model

Model stability and potential optimism were assessed using bootstrap internal validation (2000 resamples) of the multivariable logistic regression model including MOLES score and Mel*AI*noma score as predictors, with melanoma diagnosis as the outcome. In the complete-case dataset (*n* = 86), the apparent discrimination of the model was high (AUC 0.93). The estimated optimism in AUC was small (0.006), yielding an optimism-corrected AUC of 0.92. Tjur’s R^2^ decreased from 0.52 to 0.50 after optimism correction, while the Brier score increased from 0.10 to 0.11. The optimism-corrected calibration slope was 0.89, indicating modest overfitting but overall stable model performance.

### 3.8. Sensitivity Analysis by Imaging Modality

To determine if the AI algorithm’s performance remained stable across different clinical presentations and imaging hardware, a subgroup analysis was performed. Lesions were categorized based on whether they were captured with standard-field or ultra-widefield systems. It should be noted that while Mel*AI*noma scores were derived solely from these color photographs, MOLES scores were established by ocular oncologists with access to multimodal imaging (e.g., OCT and autofluorescence). In the standard-field subgroup, Mel*AI*noma achieved an AUC of 0.87 (95% CI 0.78–0.96) compared to 0.80 (95% CI 0.67–0.93) for MOLES with no statistically detectable difference between the methods (*p* = 0.42, DeLong test). Similarly, in the ultra-widefield subgroup, Mel*AI*noma achieved an AUC of 0.87 (95% CI 0.72–1.00) compared to 0.79 (95% CI 0.61–0.97) for MOLES (*p* = 0.48). These findings indicate that Mel*AI*noma maintains a high and stable diagnostic accuracy across different clinical subgroups, even when compared to an expert scoring system supported by multimodal data.

## 4. Discussion

In this study, we demonstrate that both the MOLES system and the Mel*AI*noma algorithm were associated with melanoma diagnosis in a cohort of small pigmented choroidal lesions. The two systems demonstrated a statistically significant association, and higher values of either score were linked to higher odds of melanoma. This is clinically relevant, as both approaches are primarily intended for use by non-expert opticians, optometrists, and ophthalmologists in settings where early triage of suspicious lesions is increasing demand [29,30].

Despite this overall concordance, there were notable differences between the two systems. Although the association between MOLES and Mel*AI*noma scores was statistically significant, linear regression displayed substantial dispersion, with MOLES explaining only a limited proportion of the variability in Mel*AI*noma score. This suggests that, while related, the two systems capture overlapping but not identical information. In univariable binary logistic regression, Mel*AI*noma showed slightly stronger associations with melanoma diagnosis than MOLES. This difference may partly reflect the distributional properties of the two scores: MOLES values were heavily clustered at low scores, whereas Mel*AI*noma scores were more continuously distributed across lesions, allowing for greater separation in estimated odds across the range of values.

In multivariable logistic regression, both MOLES and Mel*AI*noma scores remained associated with melanoma diagnosis after adjustment for one another, indicating that each score contributed information beyond the other. Clinically, the low shared variance between the scores (R^2^ = 0.16) means that MOLES and Mel*AI*noma will often assign different risk levels to the same lesion and therefore should not be considered interchangeable. This discordance is expected because MOLES is a feature-based ordinal score derived from multimodal assessment (including OCT and autofluorescence), whereas Mel*AI*noma is a continuous probability derived from a single color photograph. Although the statistical evidence was slightly stronger for Mel*AI*noma, the results do not suggest that one system renders the other obsolete; rather, they indicate that the two approaches capture overlapping but partly distinct aspects of lesion assessment and may provide complementary information in clinical triage.

The slightly higher explanatory power observed for Mel*AI*noma in univariable models is unlikely to be solely a statistical consequence of scale differences between a continuous probability output and an ordinal score. Discrimination analyses showed similar AUC estimates across imaging subgroups without statistically detectable differences between methods. Moreover, in the multivariable model, both scores remained independently associated with melanoma diagnosis, indicating that the association of Mel*AI*noma was not merely a reflection of statistical scaling but represented additional information not captured by MOLES.

A notable finding of this study is that Mel*AI*noma achieved numerically higher AUC estimates than MOLES despite being based solely on a single color fundus photograph, whereas MOLES scoring was supported by multimodal imaging, including OCT and autofluorescence. Although these differences were not statistically detectable, the point estimates suggest that the deep learning model captures diagnostic features from standard photography that are at least comparable to those identified by experts using additional imaging modalities. In primary care or optometric settings where OCT and autofluorescence are often unavailable, the ability to provide a reproducible risk estimate from a single photograph may therefore represent a practical advantage [31,32].

An additional practical distinction between the systems is that Mel*AI*noma provides fully reproducible output, yielding the same score each time a given image is analyzed, independent of the user. In contrast, MOLES scoring is subject to human interpretation. In the present study, all MOLES scores were assigned by experienced ocular oncologists, effectively establishing a performance ceiling for the manual scoring system. Because previous research has shown that MOLES performance can vary significantly based on observer expertise, it is probable that the diagnostic gap between the two systems would be even more pronounced in the primary care settings for which they are intended, where the AI’s consistency would remain stable while manual scoring accuracy might decline [15,19]. Non-expert performance was not evaluated in this cohort because the aim of the present study was to compare the intrinsic associations of the two scoring systems under standardized, expert-applied conditions. Evaluation of MOLES and Mel*AI*noma in their intended non-expert user groups has been addressed separately in prior work, where referral thresholds and real-world trade-offs were analyzed [15].

In terms of real-world referral thresholds, we previously evaluated explicit cutoffs for both MOLES and Mel*AI*noma in an optometrist triage setting using multimodal ocular oncologist assessment as reference [15]. Using the current Swedish Optometry Association guidance of referring lesions with MOLES score ≥ 1, MOLES achieved very high sensitivity (98%) but low specificity (17%) for melanoma classification (overall accuracy 33%). Raising the referral threshold to MOLES ≥ 3 (corresponding to “probable melanoma”) reduced sensitivity to 75% while increasing specificity to 53% (overall accuracy 57%). In the same study, Mel*AI*noma was assessed using a prespecified probability cutoff (0.63) and showed a sensitivity of 80% and specificity of 90% (overall accuracy 88%). Taken together, these data suggest that lower MOLES thresholds prioritize sensitivity at the cost of substantial over-referral, whereas a prespecified Mel*AI*noma cutoff can provide higher specificity while maintaining sensitivity in a clinically relevant range. In practice, the optimal cutoff should be selected based on the intended use case (screening vs. referral triage) and acceptable trade-offs between missed melanomas and false-positive referrals.

Another conceptual difference is that Mel*AI*noma does not explicitly base its classification on predefined clinical risk factors for growth. Although such factors are well established and clinically useful, they do not necessarily reflect underlying tumor biology, including genetic and cytogenetic alterations known to be associated with prognosis [33,34,35,36]. Whether image-based AI systems such as Mel*AI*noma capture information related to these biological features remains an open question. In future work, we will evaluate how Mel*AI*noma scores relate to genetic and cytogenetic markers, such as monosomy 3 and *BAP1* mutation status, and investigate whether image-derived features capture aspects of tumor biology beyond established clinical risk factors [35,37].

### Strengths and Limitations

This study has several limitations. First, it was conducted at a single national ocular oncology referral center, which may limit generalizability to other clinical settings, referral patterns, and patient populations. Although images were acquired using both standard-field and ultra-widefield cameras, the Swedish referral context and patient mix may not be representative of other populations [38,39]. Second, none of the included lesions underwent histopathologic confirmation, and the reference standard was therefore based on expert clinical diagnosis using multimodal assessment. Third, the use of two different fundus photography systems may have introduced variability in image properties that could influence algorithm-derived scores, although prior validation showed stable Mel*AI*noma performance across standard-field and ultra-widefield imaging modalities [19]. Fourth, genetic and cytogenetic data were not available, precluding analysis of associations with established molecular prognostic factors. Fifth, both diagnoses and MOLES scores were established by subspecialized ocular oncologists. Results may therefore differ if diagnoses and scoring were performed by non-experts, a scenario that would likely disadvantage MOLES more than Mel*AI*noma. Furthermore, MOLES scoring and the reference diagnosis were produced within the same subspecialty environment. The MOLES features largely overlap with the clinical and imaging features ocular oncologists routinely use when distinguishing small melanomas from nevi. This creates a risk of shared-feature incorporation, in which a predictor is correlated with the outcome partly because the predictor’s components are also used (explicitly or implicitly) in forming the reference classification. If anything, this would be expected to further strengthen the observed association between MOLES and diagnosis and thereby favor MOLES over Mel*AI*noma, which was generated independently from color fundus photographs alone. The fact that MOLES and Mel*AI*noma showed only modest shared variance (R^2^ = 0.16) and that Mel*AI*noma remained associated with diagnosis after adjustment for MOLES suggests that the two methods capture overlapping but non-identical information. Importantly, risk factors for growth of choroidal nevi are not necessarily true risk factors for malignant transformation, but these concepts can become conflated when the same parameters are used both to define melanoma and to predict its biology, as has been discussed previously [34,40]. Sixth, the Hosmer–Lemeshow test has limited power in small samples, and calibration results should therefore be interpreted with caution [41]. Seventh, the distribution of MOLES categories was imbalanced, with relatively few lesions in certain groups, which may reduce statistical stability and widen confidence intervals for category-specific estimates. Eighth, there was an asymmetry in the data available to each system; while MOLES scoring was supported by multimodal imaging, Mel*AI*noma was restricted to color fundus photography. Consequently, this study could not evaluate whether the addition of OCT or autofluorescence data would further enhance the algorithm’s performance. Furthermore, while the current results demonstrate the AI’s efficacy as a standalone triage tool, its integration with other clinical data remains a subject for future investigation.

The study also has several strengths. The cohort was independent of the datasets used to develop and train the Mel*AI*noma algorithm. Diagnoses were established using multimodal expert assessment, including ultrasonography, OCT, biomicroscopy, and fundus imaging. In addition, classification of lesions as nevi required at least five years of follow-up without reclassification as melanoma, increasing confidence in the correctness of the ground truth diagnoses.

## 5. Conclusions

Both MOLES and Mel*AI*noma were associated with expert melanoma diagnosis and demonstrated good discrimination in this cohort. However, statistical association does not by itself establish clinical utility, which depends on referral thresholds, decision context, and acceptable trade-offs between sensitivity and specificity. Within these constraints, the findings support their potential role in lesion triage. Clinically, a fully reproducible, photo-based system that performs comparably to expert MOLES scoring supported by multimodal imaging may be meaningful for lesion triage, particularly in settings where multimodal imaging or subspecialist expertise is unavailable. Taken together, these results support the potential role of Mel*AI*noma as a complementary aid in the assessment and triage of pigmented choroidal lesions. Future studies should include external validation in independent multicenter cohorts and prospective evaluations in real-world triage settings to confirm generalizability and assess clinical impact.

## Figures and Tables

**Table 1:** Characteristics of included patients and lesions.

*n* Images	86
*n* unique patients	86
Sex, *n* (%)	
Male	38 (44)
Female	48 (56)
Age, mean years (SD)	58 (14)
Fundus photography type, *n* (%)	
Wide field	31 (36)
Standard field	55 (64)
Nevi, *n* (%)	57 (66)
Melanomas, *n* (%)	29 (34)
Nevus LBD, mean mm (SD)	4.4 (1.6)
Nevus thickness, mean mm (SD)	1.6 (0.4)
Melanoma LBD, mean mm (SD)	6.6 (2.1)
Melanoma thickness, mean mm (SD)	2.8 (1.5)
Mean MOLES score (SD)	
Overall	1.6 (2.2)
For nevi	0.7 (1.2)
For melanomas	3.9 (2.4)

LBD, largest basal diameter. SD, standard deviation. MOLES assigns a score of 0, 1, or 2 for Mushroom shape, Orange pigment, Large size, Enlarging tumor, and Subretinal fluid, based on their absence, borderline presence, or presence. Lesions are classified as common nevi, low-risk nevi, high-risk nevi, or probable melanoma, based on their total score being 0, 1, 2, or more than 2, respectively, as described previously [21].

**Table 2:** Univariable binary logistic regression for MOLES score and melanoma diagnosis.

Predictor	Scale	OR	95% CI	*p*
MOLES score	Per 1-unit increase	2.29	1.59 to 3.50	<0.0001

Binary logistic regression with true diagnosis as outcome. CI, confidence interval. OR, odds ratio.

**Table 3:** Univariable binary logistic regression for Mel*AI*noma score and melanoma diagnosis.

Predictor	Scale	OR	95% CI	*p*
Mel*AI*noma score	Per 0.1 increase	2.27	1.57 to 4.23	<0.0001

Binary logistic regression with true diagnosis as outcome. CI, confidence interval. OR, odds ratio.

**Table 4:** Multivariable binary logistic regression for melanoma diagnosis.

Predictor	Scale	OR	95% CI	*p*
Mel*AI*noma score	Per 0.1 increase	2.82	1.62 to 6.85	<0.0001
MOLES score	Per 1-unit increase	2.24	1.40 to 4.02	0.002

Binary logistic regression including both MOLES score and Mel*AI*noma score as predictors. No interaction terms or variable transformations were included. CI, confidence interval. OR, odds ratio.

## Data Availability

Due to the sensitive nature of the clinical data, including images utilized in this study, the authors are unable to share these materials in compliance with Swedish law. The confidentiality and privacy regulations governing patient information strictly prohibit the distribution of such data.

## Abbreviations

The following abbreviations are used in this manuscript:

AI	Artificial intelligence
AIC	Akaike information criterion
AJCC	American Joint Committee on Cancer
AMD	Age-related macular degeneration
AUC	Area under the curve
CI	Confidence interval
MOLES	Mushroom shape, Orange pigment, Large size, Enlarging tumor, and Subretinal fluid
OCT	Optical Coherence Tomography
OR	Odds ratio
LBD	Largest basal tumor diameter
SD	Standard deviation
